# The impacts of political activity on fires and deforestation in the Brazilian Amazon rainforest: An analysis of social media and satellite data

**DOI:** 10.1016/j.heliyon.2023.e22670

**Published:** 2023-11-22

**Authors:** Vinicius Picanço Rodrigues, Marco Antonio Leonel Caetano

**Affiliations:** a Insper Institute of Education and Research, São Paulo, Brazil; b Deparment of Design, Manufacturing and Engineering Management, University of Strathclyde, United Kingdom

**Keywords:** Amazon rainforest, Predictive analytics, Social media, Machine learning, Political discourse

## Abstract

Social media has become a one-stop shop for consuming news and expressing political views. Politics has become increasingly emotional, and the ensuing polarization has created echo chambers that favor narratives and stories that repeat only one point of view. In this article, we investigated the role of political activity through Twitter (now ‘X’) engagement as a predictor of destructive fires and deforestation in the Brazilian Legal Amazon (BLA). We used a machine learning approach based on sentiment analysis and satellite data. To test the consistency of the sentiment analysis, we compared the timing of messages related to fire and deforestation events with daily fire data from satellites. When comparing positive and negative comments about fires in the BLA, the results showed that the best model for predicting fire outbreaks is the decision tree regressor. We found evidence that positive comments about agriculture, industry, and the Amazon rainforest in response to speeches and statements by high-ranking Brazilian politicians tend to induce positive comments about fire outbreaks and deforestation. These comments then become good predictors of fire outbreaks with a 6-day lag. These results support the view that high-ranking politicians have enormous power to influence damaging events that can have severe impacts on communities, the environment, and the economy. Brazil has seen an unprecedented increase in deforestation and fires in the Amazon rainforest in recent years. Our findings contribute to the growing literature on the role of social media in real-world events and how machine learning approaches can be used to address this class of problems.

## 1 Introduction

The Amazon region is one of the world's most important carbon sinks, containing the largest tropical forests that capture significant amounts of CO_2_. Extensive deforestation over the years has led to a reduction in its capacity for storing carbon followed by an increase in greenhouse gas emissions [1]. In particular, the Brazilian Legal Amazon (BLA) region has been experiencing a sharp increase in fire spots and deforestation since 2019, causing alarm among the international scientific and political communities [2,3]. The region is an essential component of local and global climate regulation mechanisms, providing critical ecosystem services and biodiversity [4].

Such destructive patterns have also seriously threatened the livelihoods of local indigenous peoples, whose protected areas and environmental conservation efforts are among the key factors in combating deforestation and destructive fire outbreaks in the BLA [5,6]. The combination of increasing fires and deforestation with the overarching effects of climate change poses a serious threat to the region, with experts warning that the Amazon Forest could reach a tipping point with devastating consequences, including fundamental changes in hydrological regimes and land surface temperature, soil degradation, and irreversible biodiversity loss [6,7].

In order to develop effective environmental management policies and implement conservation actions, it is crucial to gain a comprehensive understanding of the processes occurring in the Amazon. Fires and deforestation in the BLA are complex issues with multiple dimensions, including environmental, social and economic factors that play out at different spatial and temporal scales [8]. However, the political dimension is often underestimated and overlooked, despite its crucial role in shaping environmental policies and decision-making processes. The conservation of the BLA can be interpreted as a battle of conflicting interests between agribusiness and conservation efforts, with the election of Brazilian President Jair Bolsonaro in 2019 representing a major setback and the reintroduction of failed policies [9,10].

It is therefore essential to examine the political dimension of these issues and to understand how it interacts with other factors to affect the Amazon's ecological and social systems. By focusing on the political dimension of the problem, researchers and practitioners can shed light on the underlying causes of deforestation and forest fires, and provide insights for policymakers to develop more effective conservation strategies. As much of the political engagement is currently taking place in social media, we turn our attention to understanding its critical role.

### 1.1 The critical role of social media on political engagement

Social media platforms have been increasingly used for individuals to consume news about politics, and the performance of local and national governments, while simultaneously disseminating this information across their networks, reveling political affinity and preferences [11,12]. These platforms enable an interactive one-to-few or many-to-many- communication at an international scale [13,14]. Within this social media context, a large crowd of users can anonymously and cost-effectively promote their criticisms, views, and protests [15]. In informational contexts where truth seems to be either uncertain or ambiguous to the public, and the situation may contain reliable information yet unverified by trusted sources, rumors can emerge and seriously affect individuals and their communities [16].

Research on the use of social media for the promotion and sharing of news, rumors and information has found that emotional arousal facilitates diffusion, therefore making emotional content more likely to be shared and consumed by others in the network [17,18]. More particularly, scientific evidence suggests that the human brain's structures that respond to primary rewards (i.e., necessary for the survival, such as food) and secondary rewards (i.e., derived from the primary rewards, such as money) are also directly related to how people process social rewards [19].

With the wide spread of the “like” features across virtually all social media platforms, receiving many likes on a particular content and the experience of sharing information with others activates and elicits the brain's rewards structures [20]. These reward mechanisms are not only involved in the processing of subjective experiences of pleasure but also how we may recognize and respond to rewards [21]. In general, human behavior on social media is consistent with the fundamentals of reward learning as users tend to manage their posts to maximize the social rewards, while simultaneously accounting for the efforts of posting and the opportunity cost of not posting anything [22].

Since topics related to local and national politics are intrinsically emotional, the specialized literature asserts that people with strong emotional responses to political content tend to be more likely to share and consume political news and information on social media platforms [23,24]. Partisan content sharing is consistently related to anger targeted at opposing political parties and points of view, leading to greater levels of anger and dislike of the opposition [23,25,26]. Partisan social media engagement can be highly polarizing and guide attitudes and behaviors towards people and organizations with opposing views [23,[27], [28], [29]] as it only elicits anger but also evokes anger toward a specific person or topic. Anger is an approach emotion that can lead people to act [30] and is associated with increased political information sharing [23,30].

Thus, this polarization effect generates “echo chambers” or “filter bubbles” in which some people will be exposed to only one point of view, therefore only favoring narratives and stories that reiterates that particular point of view [31]. Consequently, these echo chambers tend to standardize the general attitudes and behaviors of groups around a topic that, in turn, has results in the real, physical world [32]. These results may increase societal divide, political polarization, extremism and conspiracy theories [33] as heterogeneous, contradictory claims and statements about political aspects emerge, users tend to embrace simplification to reduce complexity [13]. These polarized groups strive to differentiate themselves in the online arena by attracting and gaining more followers (audience), sticking with the group’s set of attitudes and behaviors, even if they their assumptions are wrong [13,34].

More specifically, the so-called “social bots” play a large role in the spread of misinformation, fake news, and the development of political polarization and extremists, with potentially devastating consequences [14,35]. Unidentifiable technology developers design these social bots as mechanisms to automatically engage in the dissemination of false information, often motivated by immediate financial rewards. These bots use common names and pictures to imitate genuine human identity and are programmed to continuously disseminate false news, comments and shares encompassing a pre-defined set of keywords and hashtags [13].

Some studies estimate that 9 %–15 % of all “users” in social media platforms are social bots, which are frequently unrecognizable by human users [13,36]. At this massive scale, content shared on social media that strongly appeals to the extraordinary - including blatant lies, defamation and eventually violence – maximizes the value of digital advertisements and other financial rewards. Therefore, coordinated efforts based on the widespread use of social bots can critically multiply the effect of information distortion and manipulation on social medias, thus generating serious consequences in real life [13,[37], [38], [39]].

### 1.2 The position of high-ranking politicians on social media and news outlets

As politicians's positions in social media can have serious political and economic consequences, studies have started to devote attention to understanding the context and causal mechanisms behind this complex phenomenon [40]. Between 2019 and 2022, the Brazilian Federal government and other ideologically aligned political groups have made extensive use of social media to attack science and deny important facts and practices regarding the preservation and protection of the Amazon. They also denied the increase in fire spots, even when official satellite data were publicly released by independent agencies and minimized the severe effects of these fires on climate and society at large [3,41,42].

This case is based on a reaction to the official press to these dynamics promoted by the Brazilian Federal government. As the professional media depicts and reports deforestation in the BLA region, the social media users respond in a feedback movement, with a negative sign, inverting reality. As more professional media talks about the topic, negative reactions substantially increase, creating a “wave effect”. Appendix 1 displays some of the most important international media releases on the widespread Amazon Forest's fire spots in August and September, when historical highs were reached.

In the Climate Summit 2021, Brazil's former President Jair Bolsonaro [43] affirmed in his speech on the 22nd of April: “*I determined that our climate neutrality should be achieved by 2050, anticipating the previous signaling by 10 years. Among the necessary measures, I highlight here the commitment to eliminate illegal deforestation by 2030, with the full and prompt application of our Forest Code. With that, we will reduce our emissions by almost 50 % by that date*”. One day after the speech by Mr. Bolsonaro, the president of the United States of America, Mr. Joe Biden, observed: “*We heard encouraging news announced from Argentina, Brazil, South Africa and South Korea*. *The commitments that we have made need to come true*”.

Despite the speech at the Climate Summit, Mr. Bolsonaro approved severe cuts in the budget of the Ministry of Environment [44]. When politicians use social media to issue official communication, the universe of followers not only use words for a clash of ideas, but most of the time they use those words for real action [14]. Politicians are preferring to promote fights and hatred on social media, looking for "likes" as a reward for repeating followers' ideas, even if those ideas are socially incorrect.

### 1.3 Aims and scope

Google Trends is a powerful indicator of general interest in specific subjects and topics [3]. utilized Google Trends as an indicator of actions related to fires in the Amazon during 2019, with the study discovering a 55.73 % cross-correlation between keyword searches on Google Trends and an actual increase in fire outbreaks and deforestation. However, Google Trends data do not reflect a positive or negative sentiment regarding a particular subject, as the data merely suggest an increase in interest leading to more searches for related keywords. To expand on this analysis, we examine the sentiment analysis that drives the interest in this topic and its connections to political activity as a motivator.

Despite the extensive research on environmental management, there is still a significant gap regarding studies that use sentiment analysis of political activity to investigate real-world events and actions related to environmental conservation and degradation. Particularly in the BLA region, there is a growing need to examine the impact of political activity on environmental issues as mechanisms to inform policy response and anticipate potential negative impacts on the forest and other related environmental services.

Many studies have used platforms like Google Trends or Twitter (now ‘X’) to measure public interest and engagement in environmental issues. But we still have to delve into how political opinions and sentiments shape people's actions and attitudes towards the environment, especially after exposure to high-level political discussions. By probing deeper into this area, we can uncover key insights that could have a profound impact on decision-making processes. Specifically, in the context of the BLA region, understanding this dynamic can offer a clearer roadmap for policymakers. It can guide them in crafting effective strategies for environmental management, conservation, and protection in the BLA region. Such insights can help ensure that policies resonate with the public and lead to more sustainable outcomes, therefore reverse the upward trend in destructive fires and deforestation.

Therefore, in this article, our primary focus revolves around the following research question: *To what extent can political sentiment expressed on Twitter serve as an indicator for fire outbreaks and deforestation events in the Brazilian Legal Amazon (BLA)?* Through this analysis, we endeavor to explain the potential relationship between political discourse activity and the pronounced rise in both destructive fires and deforestation within the BLA. Using sentiment analysis tools, we are able to systematically evaluate and quantify the spectrum of positive to negative intentions embedded within Twitter user activity.

Our methodology leverages a machine learning framework, which is grounded in empirical data sourced from satellite imagery and social media. This approach enables the prediction of fire outbreaks and deforestation events in the BLA, with particular emphasis on the influence of disseminated political news and official governmental stances proliferated on social media platforms. Recent examples of successful applications of machine learning techniques for time series forecasting can be found in various domains.

One study by Ref. [45] compared different machine learning models for time series forecasting and found that these models can outperform traditional methods. Another study by Ref. [46] used recurrent neural networks (RNNs), specifically Long Short-Term Memory (LSTM) networks, to forecast time series data and achieved better results compared to other methods. Similarly [47], explored the use of machine learning models, particularly neural networks, as an alternative to statistical methods for forecasting non-hierarchical time series.

In the field of energy [48], applied machine learning algorithms to forecast solar power generation based on weather conditions. They found that these algorithms yielded promising results in time series forecasting. In a similar vein, a study by Ref. [49] applied both conventional stochastic methods and advanced machine learning methods for time series forecasting in the context of hot metal temperature in steelmaking. Machine learning techniques have also been successfully applied in the financial domain [50]. explored the use of machine learning methods and new datasets to forecast US inflation. They found that these methods provided benefits in forecasting accuracy [51]. used machine learning techniques for forecasting Net Asset Value (NAV) in the financial sector and found that these techniques outperformed other statistical models.

In the field of agriculture, machine learning techniques have been applied to forecast agricultural prices [52]. used machine learning techniques to model time series data for forecasting agricultural prices, specifically focusing on brinjal in Odisha, India. They found that these techniques were successful in modeling time series data. Machine learning techniques have also been applied in various other domains such as logistics coordination [53], hydrological processes [54], and fraud detection [55]. These studies demonstrate the versatility and effectiveness of machine learning techniques in time series forecasting across different domains.

## 2 Materials and methods

### 2.1 Data acquisition system

The main rainforest area is located within the BLA region, which has a total area of 5,217,423 km^2^. This region covers nine Brazilian states: Acre, Amapá, Amazonas, Pará, Rondônia, Roraima, Mato Grosso, Tocantins and Maranhão. Fire outbreak data from INPE - Instituto Nacional de Pesquisas Espaciais (National Space Research Institute) are available with cumulative totals by month at http://queimadas.dgi.inpe.br/queimadas/portal-static/estatisticas_estados/. To compare the volatility of fire outbreaks, we chose the region of the Legal Amazon and, in particular, the State of Amazon (AM), because its area corresponds to more than 30 % of the BLA. Deforestation data were obtained from INPE and are available on http://terrabrasilis.dpi.inpe.br/app/map/alerts?hl=pt-br using the DETER system.

Twitter allows searching for tweets based on recent searches and searching the entire archive. Both variables involve using a single search query to filter tweets around a specific topic. These queries are created using a set of operators that match tweets and user attributes, such as message keywords, hashtags, and URLs. Operators can be combined into queries using Boolean logic and parentheses to refine the matching behavior of the queries. We used a Python library called Tweepy, which allows you to use the API with Python.

**Fig. 1:**
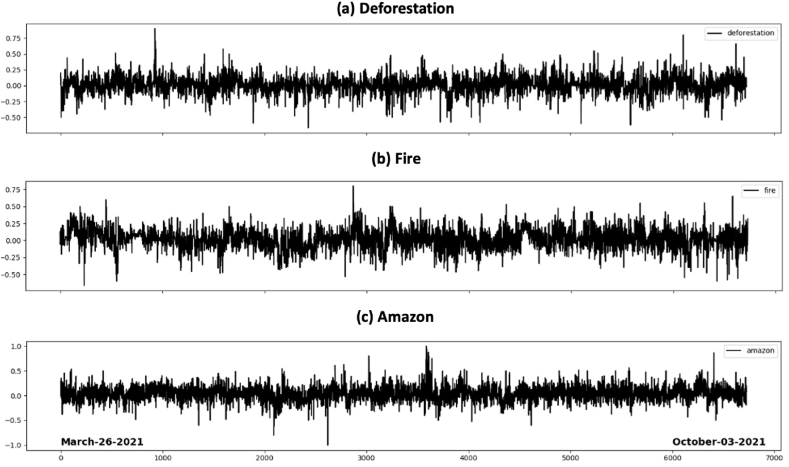
Data of Sentiment Analysis from Twitter for keywords **(a)** “desmatamento” (deforestation), **(b)** “queimadas” (fire) and **(c)** “amazônia” (Amazon).

With Tweepy and the authorization of Twitter for developers with *consumer_key*, *consumer_secret, access_token and access_token_secret*, we built scripts in Python to collect data by searching for keywords related to deforestation and fire events in BLA. In this library, there is a function called “search” where you enter the keyword and choose the desired number of messages and the period. Thus, the script acquired the last 5 messages every 20 min from more than 20 keywords. Using the “re” library, we filtered out all unwanted symbols, such as @, #, & and placed only the words from the messages in a vector. Twitter sentiment analysis uses English as the default language to measure comments using scores. Since tweets, comments, and content from Brazil are typically written in Portuguese, we used the GoogleTrans library to translate Portuguese comments into English, removing punctuation, commas, and hashtag symbols from tweets. This vector of words was also analyzed after the captures, and the sentiments were calculated using the *analysis. sentiment.polarity* function from the Tweepy library. After each word from these 5 messages that were in the vector received the sentiment values calculated by Tweepy, the script calculated the average sentiment of the keyword (formed by all related words in the vector). This average value is saved in a database in Excel spreadsheet format. Fig. 1 shows the acquired data from sentiment analysis of Twitter using Tweepy Python library between March and October 2021.

**Fig. 2:**
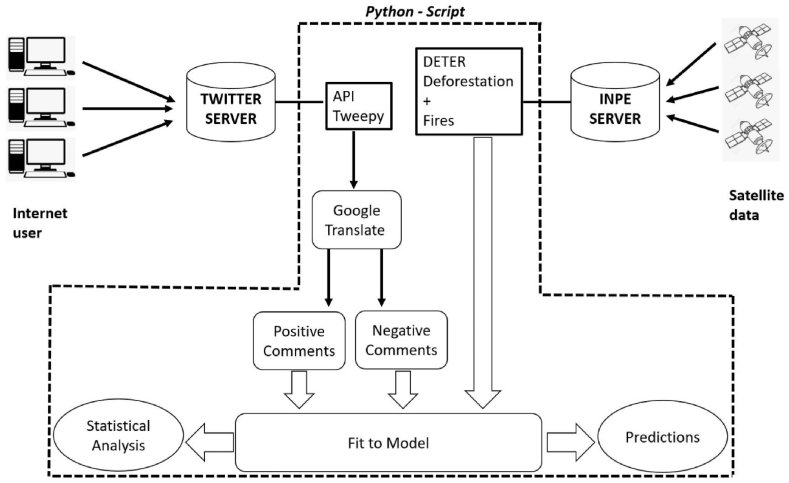
Scheme of data base acquisition.

Between 2020 and 2021 (until October), we acquired about 60,000 data points of sentiment analysis on the keywords “desmatamento” (deforestation), “incêndios" (fires) and “amazônia" (Amazon). These data have oscillation of comments measured by Twitter with minimum value −1 and maximum value + 1. We divided the comments into two classes (positive and negative sentiment). In some measures, we used the sum of comments separated by day, in other measures, we used the frequency count of positive and negative comments per day. This was done in order to adapt to the actual data of INPE, which are available daily, based on satellites that cover the entire Brazilian territory. We built a programming system that searched the data for Twitter and transformed the sentiment analysis into a signal describing the behavior on keywords (Fig. 2).

### 2.2 Negatives tweets for keyword “amazon” and influence in deforestation area

**Fig. 3:**
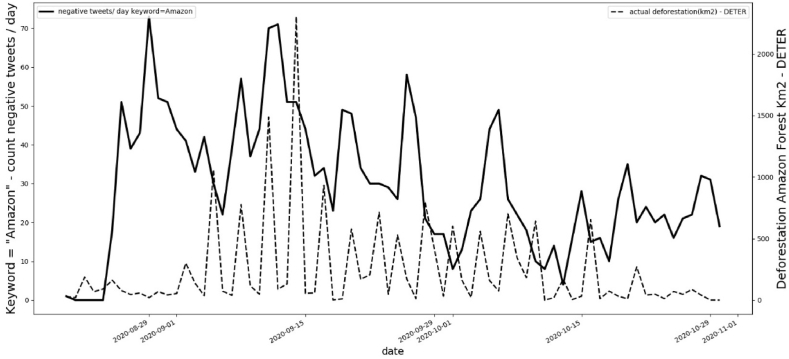
Comparison negative comments with keywords “Amazon” in Twitter and actual deforestation area in km^2^ by DETER – INPE.

Data were collected daily between August 20th-2020 and October 31st-2020 for deforestation area in Km^2^ using the DETER system of INPE. In the same period, comments in Twitter were automatically collected for every 20 min using API Tweepy with Python and separated into positive and negative messages with keyword = “Amazonia” (Amazon). Then the frequency for positive and negative posts related to “Amazon” was calculated daily. Fig. 3 shows a comparison between the daily frequency of negative messages for “Amazon” and the actual deforestation area (km^2^) measured by the DETER system of INPE.

It is possible to note that in the same period the negative comments about “Amazon” start before the days when the data on deforestation area are collected by INPE, showing a daily increase as the negative posts in Twitter increase. After October 10th, 2020, the negative messages decrease and the deforestation area decreases drastically.

**Fig. 4:**
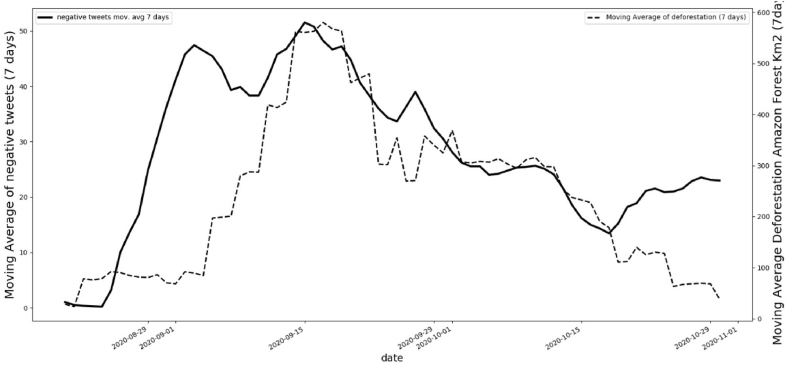
Smoothed data using moving average (7 days) for negative comments with keywords “Amazon” in Twitter and actual deforestation area in km^2^.

The relationship between negative comments about “Amazon” and actual deforestation is better observed when the moving average is calculated for the two data sets. Fig. 4 shows in the continuous line the behavior of negative comments and in the dotted line in correspondence for these messages, in the same period, the actual data for deforestation in legal Amazon in km^2^. As observed before, it is clear in this graph that when negative comments decrease, deforestation also decreases.

**Fig. 5:**
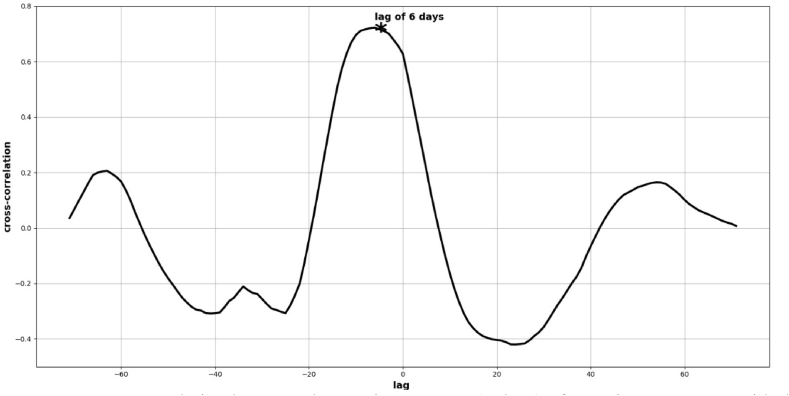
Cross-correlation between the moving average (7 days) of negative comments with the keyword “Amazon” on Twitter and the moving average (7 days) of actual deforestation area in km^2^ with a lag of 6 days.

To discover the statistical relationship between the increase or decrease of comments and the actual data of deforestation, we calculated the cross-correlation for these two data sets. The results showed (Fig. 5) that the correlation between negative comments on “Amazon” in Twitter and deforestation area in km^2^ is 72.22 % with a lag of 6 days.

It is noteworthy that there is a 6-day lag between what people say negatively about the Amazon and what happens in terms of deforestation in the region. When messages with hateful content are conveyed, the events occur about a week later. On September 22, 2020, Bolsonaro gave a speech in which he claimed that the Amazon “is humid and does not allow fires to spread” (Yahoo [56]. A few days after the speech, on September 24, Mr. Bolsonaro and Mr. Salles (the Minister of the Environment) reiterated that the forest is wet and that its central areas are not on fire. The problem, they argued, was limited to the areas surrounding the forest (O [57]. Before this speech, Twitter's sentiment index was decreasing (Fig. 3), with our script registering 29 negative points and 68 positive points of sentiment for the keyword “Amazon”. However, two days after the speech, the sentiment index changed to 58 negative points and 39 positive points (September 26th), and then to 47 negative points and 41 positive points the following day (September 27th). Fig. 3 shows the spike after the impact of this presidential discussion on the same dates.

This 6-day lag refers to the data acquired in 2020, but from 2018 to 2022, deforestation and fires in the Amazon were daily topics in Brazil. Every week the scenarios changed, and polarization increased, bringing the environmental theme to the center of political discussion in Brazil. For example, on August 11, 2019, the city of Altamira in the BLA was struck by one of the largest forest fires in history, instigated by groups on social media. This period became known as the “fire day”, where police investigation data points to a planning of fires a week before the event [3], exactly within the 6-day lag as the lag found in this 2020 data with Twitter. In our previous work [3], other cases are presented where the discussion related to the degradation of the Amazon region indeed leads to consequences a week later, some so severe that they have not been resolved to this day, such as President Bolsonaro's refusal to accept $80 million from Germany for the Amazon Fund.

### 2.3 Relationship between positive and negative tweets for keyword “queimadas” (fire) and actual outbreak fires

From March 3, 2021 to September 16, 2021, we collected data from Twitter using the keyword “queimadas” (fire in English). These data were automatically retrieved every 20 min. See Fig. 2. The number of fire outbreaks is collected and published daily by INPE using satellite imagery. To compare these two datasets, we grouped the data by day and separated the posts into positive and negative comments. In Tweepy, positive comments have a value between 0 and 1 in sentiment analysis, and negative comments have a value between 0 and -1. For example, comments encouraging fires appear with a positive sign, while comments criticizing and defending the forest and punishments take on negative values. We select the data outbreak fires from Legal Amazon in the data from INPE, which are daily counts of new fires.

**Fig. 6:**
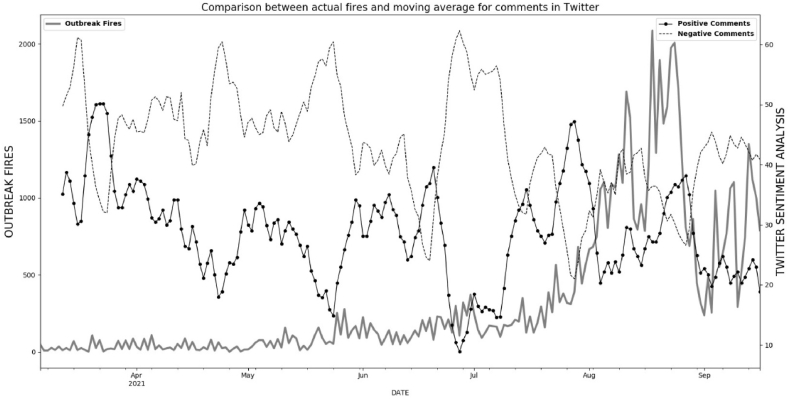
Comparison between actual fire outbreak data (INPE) and the moving average of positive (-o-) and negative (---) comments in Twitter using the “queimadas” (fire) keyboard.

Fig. 6 shows the moving average (7 days) of the daily frequency of positive and negative comments and the comparison with outbreak fires (number of fires per day). It can be seen that when positive comments about fires outnumber negative ones, the number of fires seems to increase.

For example, in July/2021, the comments supporting and encouraging fires in Brazil outnumbered the negative and opposing comments by far. What can be seen immediately afterwards is a daily increase in the number of fires registered by INPE. However, a specific type of keyword or comment alone does not determine an increase in fire movement. Other factors are driving and influencing the growing number of fires in Brazil. These include economic factors, education, and government policies. Therefore, we collected other keywords to cross-reference information about the direct influence on the increase of fires.

### 2.4 Statistical relationship between fires, deforestation, amazon, industry, and agriculture

**Fig. 7:**
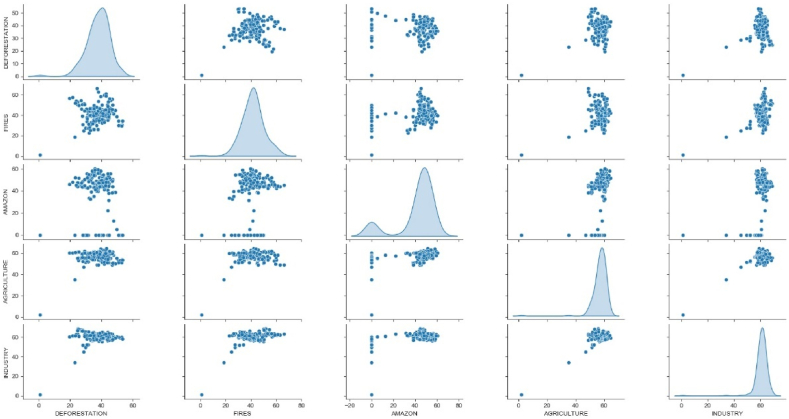
Statistics influence among keywords “desmatamento” (deforastation), “queimadas” (fires), “Amazônia” (Amazon), “agricultura” (agriculture) and “indústria” (industry). Labels are positioned along the vertical and horizontal axes, forming panel combinations that display the influences.

In order to assess other comments on Twitter that could influence the increase in burning or deforestation in the Amazon rainforest, we collected other keywords for the same period: “desmatamento” (deforestation), “queimadas” (fires), “amazônia" (Amazon), “agricultura” (agriculture), and “indústria" (industry). Fig. 7 shows a comparison between count and trend for keywords with positive comments only.

The probability distribution of positive comments per day is not a Gaussian distribution. Thus, the fat tail appears in all keyword sets and it is possible to observe the positive influence among all keywords. For example, if we highlight the data between “queimadas” (fires) and “desmatamento” (deforestation), the scatter plot shows a positive trend, which means that when there is an increase in positive comments for fires, there is also an increase in positive comments for deforestation. The same happens with the association between the word “industry” and the word “deforestation”. To reinforce this influence on positive comments for destructive events in the Amazon rainforest, we calculated the cross-correlations between these keywords.

**Fig. 8:**
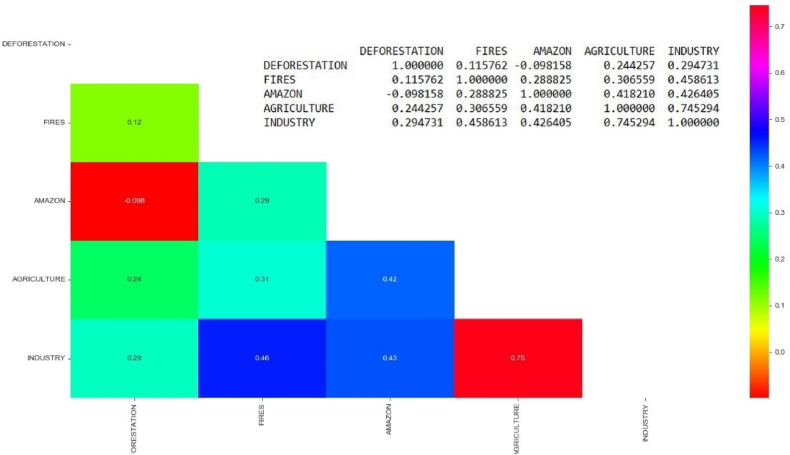
Cross-correlation between all keywords.

Fig. 8 shows the cross-correlation between all keywords and their main relationships become clearer, such as the correlation between BLA and agriculture, which presented a value of 0.41 (41 %). The relationship between positive comments for industry and BLA showed a correlation of 0.42 (42 %). The correlation between agriculture and fires was 0.30 (30 %), while for agriculture and deforestation it was 0.24 (24 %). This correlation shows that positive comments about agriculture, industry and the Amazon forest tend to induce positive comments about fire outbreaks. The positive comments about industry have a correlation of 0.45 (45 %) with comments encouraging fire outbreaks.

**Fig. 9:**
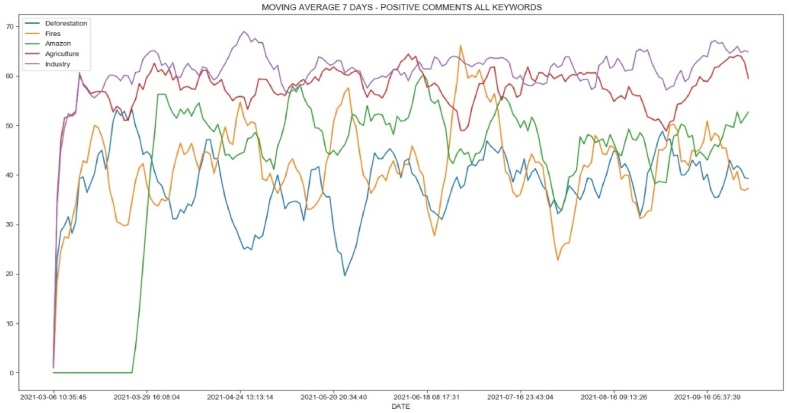
Moving average of 7 days for positive comments of keywords.

**Fig. 10:**
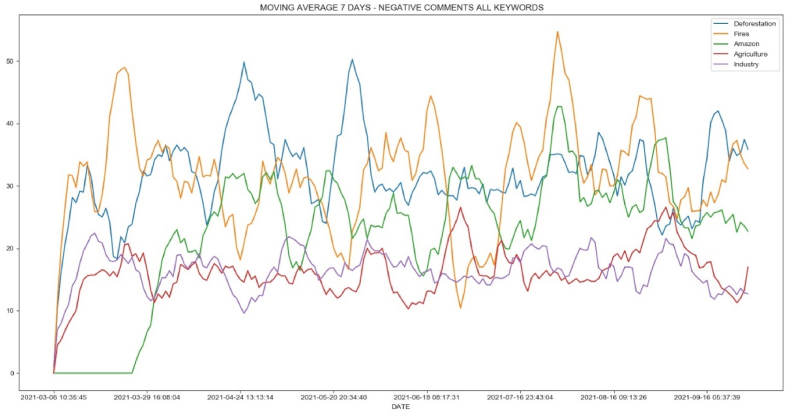
Moving average of 7 days for negative comments of keywords.

**Fig. 11:**
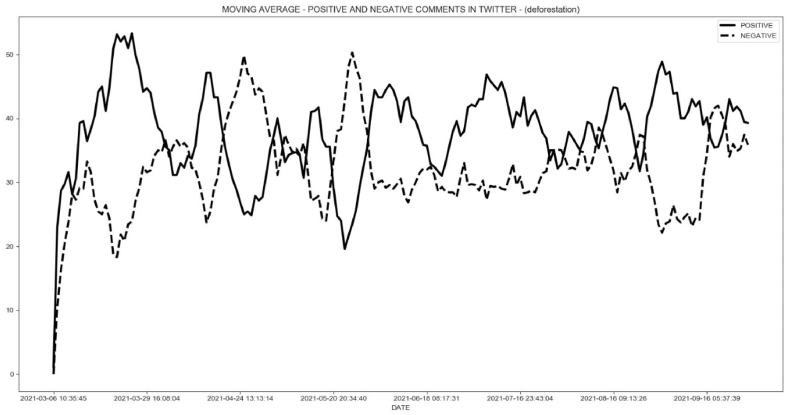
Daily comparison for positive and negative comments for keywords “desmatamento” (deforestation).

Fig. 9 shows a moving average based on daily data for positive comments using the keywords in Twitter. As noted in the previous trend and correlation statistics, similar behavior occurs when comparing the daily evolution of comments. A special case is the line graph for “queimadas” (fires) and “desmatamento” (deforestation). Towards the end of the observation period, when all positive comments are in an upward trend, positive comments about fires and deforestation start to decrease. This is because negative Twitter comments outnumber positive ones (Fig. 10, Fig. 11).

During this time, there was a constant discussion about deforestation and fires in national newspapers and television channels, with daily criticism of forest destruction in Brazil. As a result, not only did negative comments about deforestation and forest fires increase and outnumber those in favor of deforestation, but the actual data began to reflect critical behavior.

**Fig. 12:**
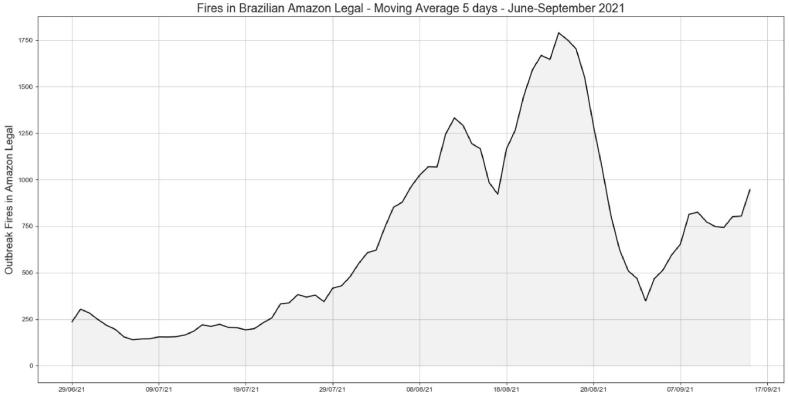
Actual data for outbreak fires from INPE.

Fig. 12 shows that after the negative comments were higher than the positive comments about fires and deforestation, the fire spots started to decrease. The spots decreased from 1750 fires on August 21st, 2021 to 300 in the same period when the negative comments were higher than the positive comments about the destruction of the BLA.

## 3 Results and discussion

The statistical results show a relationship between Twitter comments and deforestation data in the BLA. The conflict between positive and negative comments obtained by sentiment analysis of Twitter can disrupt important events related to the environment. These comments are often generated by robots with the explicit purpose of supporting a cause or supporting the actions of important government officials.

Actual data on fires and deforestation from satellites prove the trend of events, but they only show the results of decisions that have already been made. Time series analysis helps predict seasonal data with good quality, but this is only possible if the model adopted has explanatory variables and strong correlation. As shown in the previous sections, comments on deforestation and fire are related to and driven by other types of comments, such as keywords related to industry and agriculture.

A growing, trendy approach that is replacing more traditional time series analysis tools is machine learning. Artificial intelligence methods can learn from historical data and develop parameters and variables to increase predictive power. In the next subsections, we investigate the predictive power of classification and regression models for predicting fire locations based only on Twitter comments.

### 3.1 Machine learning 1: classification model of fires outbreaks

Two broad classes of methods are used in machine learning. The first large class refers to methods for sorting data. Within classification, many methods are used to help separate data and make decisions. We used the SVC (Support Vector Classification) model to determine whether the fire outbreaks were controlled or uncontrolled. We assumed that outbreaks were controlled if they were below 300 and otherwise uncontrolled. This value was assumed because in the wettest and rainiest months, the outbreak values are below 300, as can be seen in Fig. 6, on the left axis of the graph. Based on the INPE data on fires and positive and negative comments with the keyword “queimadas” (fire spots), we built a database with the following structure.

- First column has word “controlled” if outbreak fires is below 300 and “uncontrolled” otherwise.
- Second column has number of negative comments in Twitter for keyword “queimadas” (fire) daily.
- Third column has number of positive comments in Twitter for keyword “queimadas” (fire) daily.
- Fourth column has number of outbreak fire in the same day of comments in Twitter.

**Fig. 13:**
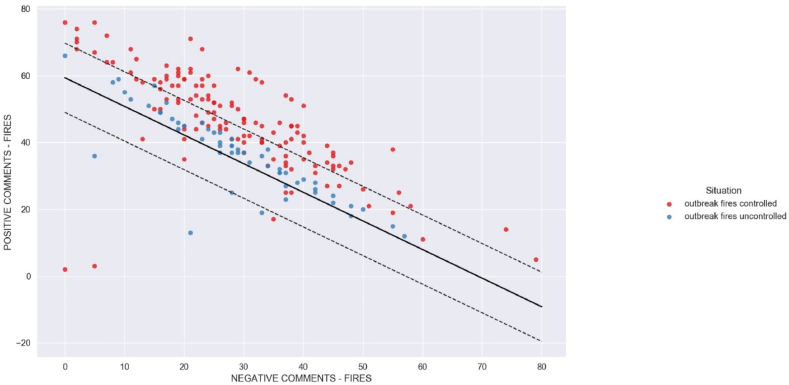
SVC algorithm of machine learning to classify outbreak fires.

The goal is to use the SVC algorithm to train a model that can return the potential number of fire outbreaks based on the number of positive and negative comments as input. Using Python's scikit-learn library, we obtained the results shown in Fig. 13.

The training and testing of the SVC algorithm yielded an accuracy of 0.7113 (71 %) for this data. In Fig. 13, the red dots are controlled outbreaks (below 300) and the blue dots are uncontrolled events. The dashed line represents the 95 % confidence interval, but this approach did not perform well when we introduced untested values. This shows that this classification problem is non-linear and more complex. Table 1 shows that the decision was biased and did not hit a true uncontrolled situation for the fires.

**Table 1:** Machine learning for SVC algorithm with linear model.

Actual situation	Negative comments	Positive comments	Prediction situation
controlled	37	33	controlled
controlled	17	53	controlled
controlled	27	44	controlled
controlled	22	48	controlled
controlled	24	45	controlled
controlled	29	41	controlled
uncontrolled	42	28	controlled (*)
controlled	38	32	controlled
uncontrolled	55	15	controlled (*)
uncontrolled	48	21	controlled (*)

### 3.2 Machine learning 2: classification model of outbreak fires with NuSVC

Since the classic SVC algorithm with linear models did not produce satisfactory results, we decided to use the NuSVC model from the library Sklearn of Python accessible in the website sklearn. svm.NuSVC in the host of scikit learn:

https://scikit-learn.org/stable/modules/generated/sklearn.svm.NuSVC.html.

**Fig. 14:**
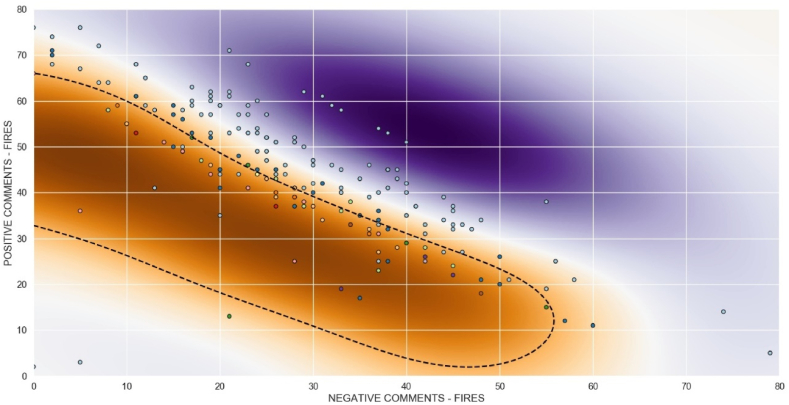
NuSVC algorithm of machine learning to classify outbreak fires.

NuSVC and SVC are similar methods that accept slightly different sets of parameters and have different mathematical formulations. A new parameter *ν* (Nu) is introduced to control the number of support vectors and margin errors with υ∈(0,1]. We adopted *ν* as 0.5 in all situations for training and testing, and following the recommendation of the scikit-learn guide. Fig. 14 shows the border of classification for fire outbreaks in the BLA between March 3rd, 2021 and September 16th, 2021.

The classification results were much better than the SVC model. Not only in terms of graphical visualization, but also in terms of accuracy in training and testing, as the accuracy was 0.93 (93 %) and 0.81 (81 %) for controlled and uncontrolled fire outbreaks, respectively.

To test the NuSVC, we used the same values of Table-1 as input for actual fire outbreaks and comments in Twitter with the keyword “queimadas” (fires). Table 2 shows the results obtained. For three uncontrolled fire situations, using only positive and negative comments from Twitter as input, NuSVC was correct in 2 of the 3 real situations. For the controlled situations, NuSVC failed to predict only one.

**Table 2:** Machine learning for SVC algorithm with linear model.

Actual situation	Negative comments	Positive comments	Prediction situation
controlled	37	33	uncontrolled (*)
controlled	17	53	controlled
controlled	27	44	controlled
controlled	22	48	controlled
controlled	24	45	controlled
controlled	29	41	controlled
uncontrolled	42	28	controlled (*)
controlled	38	32	uncontrolled (*)
uncontrolled	55	15	uncontrolled
uncontrolled	48	21	uncontrolled

Correctly predicting a controlled or uncontrolled fire situation is very important, and it is not practically available to experts in the field using only traditional time series analysis methods. As a complement to predicting whether a fire outbreak is controlled or uncontrolled, we test another machine learning approach to predict the number of fires in the future when there is no real data from satellites.

### 3.3 Machine learning 2: decision tree regressor for prediction of outbreak fires

In order to predict not only the situation of fires for the legal Amazon, but also the number of outbreaks when there is no satellite data yet, we tested machine learning for the algorithm Decision Tree Regressor. For this new methodology, we created an Excel spreadsheet that is different from the previous methodology. The first column is the daily number of fires, the second column is the daily number of positive comments, and the third column is the number of negative comments. All comments are for the keyword “queimadas” (fire). The period is also the same and the actual data are also from Legal Amazon. We assumed 90 % to train the tree and 10 % to test and maximum depth of the tree equal to 25 and minimum sample split as 2.

**Fig. 15:**
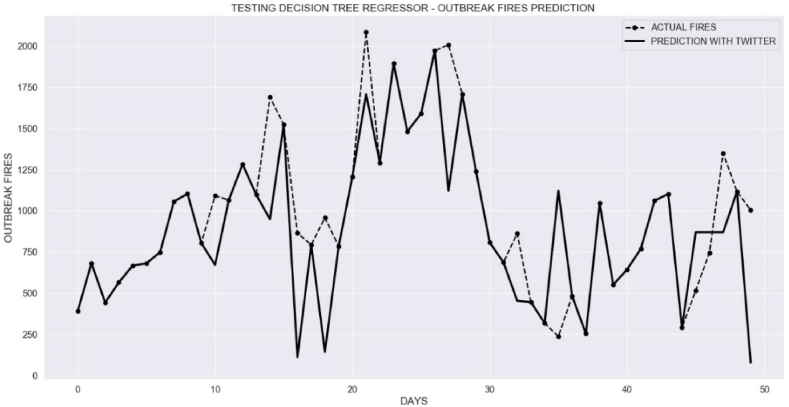
Decision tree regression algorithm for the classification of fire outbreaks in the Legal Amazon ((--) actual fires, (−) prediction).

Fig. 15 shows the result of comparing the actual data and the prediction of the Decision Tree Regressor model for fire outbreaks. The input to the model after training was the number of positive comments and the number of negative comments related to fires. The dashed line represents the actual data from INPE based on satellite imagery. The solid line is the prediction of fire outbreaks using comments only.

We calculated the root of square mean using numpy library of Python with the following formula.

(a) mean_squared_error = np. mean ((y_test.values - modelo. predict (X_test))**2)
(b) rmse = np. sqrt (mean_squared_error)

In this case, X_test is an array of data for testing the original samples from the Excel spreadsheet with positive and negative comments. The rms was 457.37 for the results observed in Fig. 5.

As shown in Fig. 5, we found the delay of 6 days between tweets about keywords “amazônia" (amazon) and the actual outbreak of fires. To improve the prediction of fires, we introduced a new table with entries of positive and negative sentiments for keyword “queimadas” (fires) with a lag of 6 days. Table 3 is an example of this 6-day lag between actual fires and sentiments in Twitter.

**Table 3:** Decision Tree Regressor with lag of 6 days.

Date (Sep - 2021)	Negative comments (fires)	Positive comments (fires)	Actual Outbreak Fires
06	13	41	–
07	33	58	–
08	55	38	–
09	56	25	–
10	37	54	–
11	21	71	–
12	31	61	11
13	38	52	25
14	23	68	11

On September 6, our script reported 13 negative and 41 positive comments about fires. To train the decision tree regressor, we selected entries [13, 41, 11], then [33, 58, 25], and then [55, 38, 11], and so on until we covered the entire dataset. Then the data for the comments can predict events for fires 6 days in the future to train the machine learning algorithm.

We chose data from March 6 to September 15, 2021, to train the machine learning algorithm based on a 6-day lag. After training, we entered 10 new data points for positive and negative comments on Twitter related to the keyword “queimadas” (fires). Then, we compared the predictions for 6 days in the future for fire outbreaks from the decision tree regressor with the actual fire data from INPE.

**Fig. 16:**
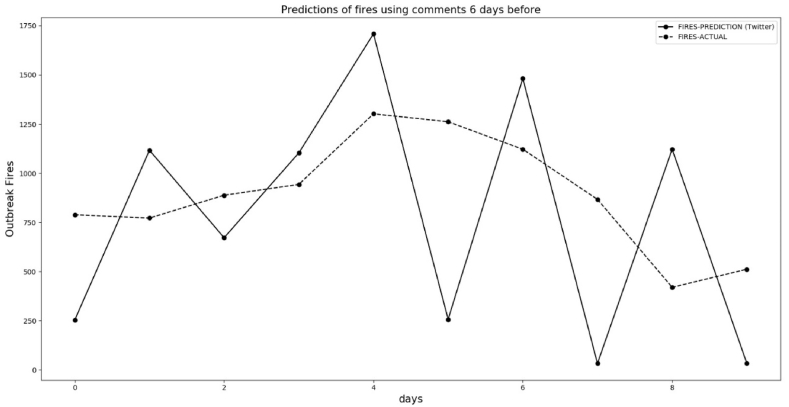
Predicted and actual fires between September 16, 2021 and September 25, 2021 with 6-day lag.

We predicted fires outbreak for 10 days considering the 6-day lag of the message from Twitter for positive and negative comments about fires. Fig. 16 shows the comparison between predictions of fires between September 16 and September 25, 2021 using historical data (September 10 to September 19). Fig. 16 shows the correct prediction trends from the Decision Tree Regressor 6 days before the actual trend of fires.

**Fig. 17:**
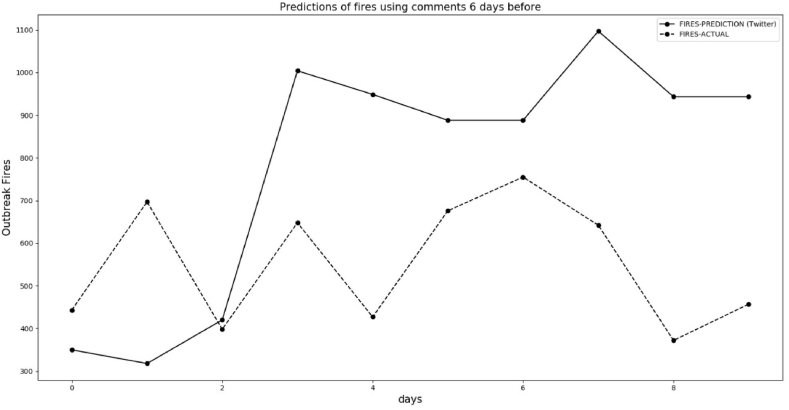
Predicted and actual fires between September 26, 2021 and October 6, 2021 with 6 days delay in reporting.

**Fig. 18:**
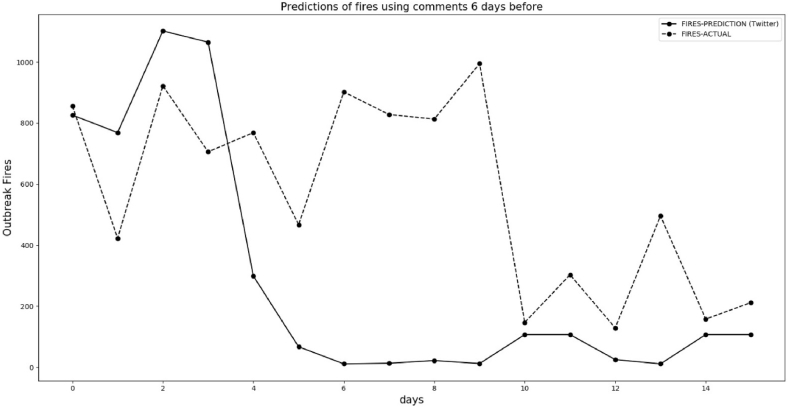
Predicted and actual fires between October 7 and October 22, 2021, with a 6-day lag.

Fig. 17, Fig. 18 follow the same procedure we've outlined, based on the 6-day lag to predict. The predictions in Fig. 17 are not satisfactory, but in Fig. 18 the machine learning algorithm anticipates a trend of decreasing fires. The explanation for the not so good result in Fig. 17 is that our dataset has less than one year in comments. Although we have many data for each day, we do not have more than one year to train machine learning. The period of Fig. 17, Fig. 18 is when the rainfall regime is increasing in the Amazon forest, and our data used to train this machine learning starts in March 2021.

To obtain better results to train the machine learning algorithm for the end of September, it is necessary to have this data of comments for one year before, but we do not have. As it is possible to observe in Fig. 18, some days in October using data with less fires, the results are better than end of September. Again, the trends of the comments show 6 days before the decrease of fires in the BLA, during the beginning of the rainy period of 2021/Oct/7 and 2021/Oct/22.

### 3.4 Long-term prediction

Using machine learning to make predictions and comparing these results with daily satellite data provides value on its own. However, this approach only verifies the efficiency of a given algorithm. To transform the outcomes from machine learning into fire predictions without relying on satellite data, we utilized optimal parameters for training. We also extended our predictions to forecast several days in advance, while maintaining consistent and unaltered learning parameters. We acquired data from Twitter from November 24, 2021 to March 15, 2022 and computed the daily frequencies for negative and positive sentiment. Without satellite data from INPE, we ran the algorithm daily to predict 6 days of fire outbreaks.

After the predictions, we acquired actual satellite data for fires and compared the predictions with actual fires. There is an important time lag between social media comments and actual events. We also used a 6-day moving average for comparison. Table 4 displays the results of the first days.

**Table 4:** Long-term prediction with lag of 6 days.

Day	Prediction (fires)	Actual (fires)	6-day Moving Average for Prediction	6-day Moving Average for Actual Data
Nov/24	195	163	195	163
Nov/25	215	113	205	138
Nov/26	41	84	150.33	120
Nov/27	41	107	123	116.75
Nov/28	98	365	118	166.40
Nov/29	543	89	188.33	153.50
Nov/30	16	183	159	156.83
Dec/01	215	126	159	159
Dec/02	37	169	158.33	173.16
Dec/03	21	50	155	163.66
Dec/04	18	37	141	109
Dec/05	215	26	87	98.50
Dec/06	21	29	87.83	72.83
Dec/07	25	137	56.16	74.66
Dec/09	76	235	62.66	85.66
Dec/10	76	76	71.83	90
Dec/11	16	91	71.50	99
Dec/12	215	30	71.50	99.66
Dec/13	14	15	70.33	97.33
Dec/14	72	169	78.16	102.66

**Fig. 19:**
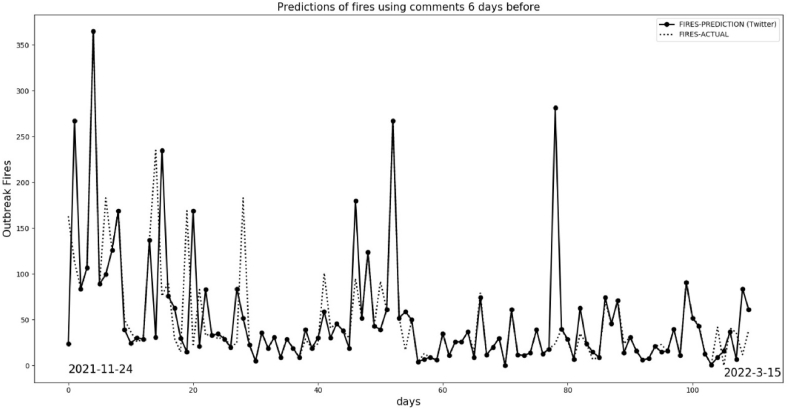
Prediction and actual fires for long time maintain fixed parameter of training.

Fig. 19 shows the results for the entire period. This figure presents a visualization of the trends (6 days ahead for each day of comments). It is impossible to get the correct values for fires, but the trends have a good concordance, keeping the same parameters without feedback of actual data from satellites. However, we claim that people's sentiment on Twitter is a reliable predictor of real actions and events in the case is the fires in the BLA.

**Fig. 20:**
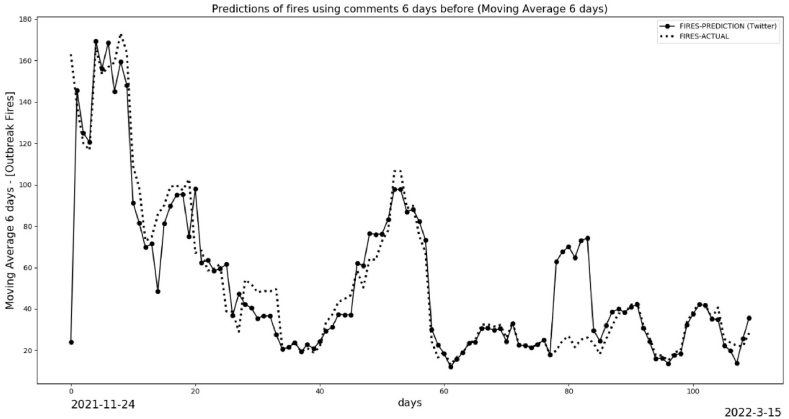
Prediction and actual fires with Moving Average 6 days.

Fig. 20 shows the 6-day moving average, which has improved results compared to Fig. 19 due to the smoothing of data. Some peaks, indicating significant differences, occurred because new posts about “Amazon” shifted the trends. These new aspects had not been previously trained by the machine learning model. It's important to note that the results in both Fig. 19, Fig. 20 were based on the same initial training data and were not updated with new data.

## 4 Conclusion

In the introduction of our paper, we outline the research question we aimed at responding: *to what extent can political sentiment expressed on Twitter predict fire outbreaks and deforestation events in the Brazilian Legal Amazon (BLA)?* This question emerged as both timely and significant in an era where politics has become emotional and polarized. Understanding how the spread and influence of political sentiment translates into real-world events has become fundamental in a society with ubiquitous social media.

This research question is fundamentally interdisciplinary. It bridges the fields of political science, environmental science, and data science. By combining sentiment analysis with satellite data, our study proposes a novel methodological approach to predict harmful events in the BLA. The potential implications of our findings could reach far beyond academic realms, especially for policymakers and activists. Establishing a link between political sentiment on social media and tangible environmental harm underscores the profound influence high-ranking politicians wield through the narratives they promote. This insight might serve as a valuable tool for governments, NGOs, and international bodies, guiding them to monitor and potentially counteract these narratives to prevent further environmental degradation.

Moreover, while various studies have delved into gauging public interest in environmental matters, our research endeavors to fill a discernible gap. We aim to go beyond mere interest, probing how sentiment—particularly political sentiment—shapes and influences attitudes and actions concerning the environment. This innovative approach, which amalgamates Twitter sentiment with real-time satellite data, potentially introduces a predictive tool that has hitherto been absent in the literature. Our research question seeks to illuminate the intricate interplay between political discourse, public sentiment, and consequential environmental outcomes. Given the increasing urgency of environmental challenges globally, understanding this nexus is of paramount importance.

Social media emerged as a tool for bridging the communication gap between people and connecting diverse interests. Initially, it was thought that this could lead to a more connected world through the sharing of ideas and experiences. However, we have observed that hate speech, discrimination, and incitement have increased with social networking. Our work explored the possibility of predicting fire outbreaks in the BLA region based on social media behavior using Twitter's sentiment analysis tool. The Brazilian government under President Jair Bolsonaro has seen an unprecedented increase in deforestation and fires in the Amazon rainforest in recent years. To test the consistency of Twitter's sentiment analysis, we compared the timing of news related to actual fire events provided by established media outlets with events and real data from INPE satellites, which report real fire outbreaks on a daily basis.

We showed that the correlation between the BLA and agriculture was 0.41 (41 %). The relationship between positive comments for industry and the BLA showed a correlation of 0.42 (42 %). The correlation between agriculture and fires was 0.30 (30 %), and between agriculture and deforestation was 0.24 (24 %). These correlations show that positive comments about agriculture, industry, and the Amazon forest tend to induce positive comments about fire outbreaks. The positive comments about industry have a correlation of 0.45 (45 %) with comments that encourage fires.

To predict the increase or decrease in the number of fires in the BLA based on a sensitivity analysis, we used machine learning algorithms to create artificial intelligence models, using as input only positive and negative comments on Twitter regarding the word “fires”. The output of the models was always quantitative, i.e. the number of fire spots. These data were then compared with actual INPE satellite data to verify the degree of accuracy.

We found that the best model for predicting fire outbreaks using Twitter sentiment analysis is the.

Decision Tree Regressor. In terms of forecasting, we show that the best methodology is to work with a 6-day lag. Thus, the data on positive and negative comments on Twitter gives better results in terms of predicting fires within 6 days after these comments appear on Twitter. We achieved good agreement in predicting fire outbreaks (see Fig. 19) by keeping the training dataset unrefreshed for about 21 days, using only the sensitivity of positive and negative comments as input to the model.

The words of politicians today have an enormous power of influence, not only in terms of digital and harmless hatred, but as we have shown in our work, in terms of real harmful effects. These real events affect the population, the environment and the people living in the BLA region and beyond. Driven by hatred through social networks, words become real weapons. However, as we have shown, we can predict the negative effects and control in advance the trend of events that could lead to the destruction of an extremely important and precious region, which for this work is the Amazon forest.

This work is not without limitations. Central to these is our focus on a single social media platform, Twitter, as the basis for our analysis. While Twitter (now ‘X’) remains a popular medium for political discourse, it has a specific set of demographic and content biases that may not capture the full range of political sentiment in broader society. The period of our data collection, spanning 2020 to 2021, was also marked by a combination of unparalleled global events. From the ongoing ramifications of the COVID-19 pandemic to political challenges in Brazil, climate crises, political upheavals, and disruptions in various industries, the background against which our study unfolds is highly complex. The influences of these events create a context that may have nuanced implications on our results, the full scope of which remains difficult to quantify. Additionally, our analysis is tied to a specific set of keywords, chosen based on extant literature. While these choices were informed, alternate methodological decisions could yield distinct, albeit related, outcomes. It is also important to note that after the data for this study was collected, Twitter (‘X’) has had major changes in ownership and strategy, followed by new data, privacy, and API policies. There are indications that crawling and scraping will be significantly limited under the X's new terms of service [58]. Since this is an ongoing process, it remains unclear how researchers will use the new data integration and what data will be made available.

Some potential future research stream can be devised. Further analysis could explicitly consider the actors involved in the social media discussion (e.g., number of followers, centrality) and the level of engagement (e.g., replies, reposts, messages). Also, diversifying sentiment analysis by incorporating varied combinations of strings and enlarging the scope to other social media platforms could offer a more holistic understanding of political engagement dynamics. Furthermore, envisioning a computational system that continuously gathers and scrutinizes data from both social media and satellites stands as a promising prospect. Such a system could act as a real-time monitor, highlighting emergent trends and serving as a tool for both long-term policy formulation and immediate interventions. Further enriching the research methodology by adopting mixed methods would also be a worthwhile exploration. By bringing in qualitative techniques from disciplines such as sociology, anthropology, and political science, future studies can delve deeper into the multifaceted nature of the observed phenomenon, unraveling layers that quantitative data alone might overlook.

## Data availability statement

Data will be made available on request.

## CRediT authorship contribution statement

**Vinicius Picanço Rodrigues:** Writing – review & editing, Writing – original draft, Validation, Project administration, Formal analysis. **Marco Antonio Leonel Caetano:** Writing – original draft, Software, Resources, Methodology, Formal analysis, Data curation, Conceptualization.

## Declaration of competing interest

The authors declare that they have no known competing financial interests or personal relationships that could have appeared to influence the work reported in this paper.

## Figures and Tables

**Table:** Table - Headlines in international media outlets reporting Amazon Forest's widespread fire in August and September 2020.

Date	Headline	Media Outlet	Link
2020-Aug-18	Amazon's widespread fire damage ‘invisible to our eyes'	DW	https://www.dw.com/en/amazon-rainforest-fire-deforestation-mining-slash-and-burn-brazil/a-54486689
2020-Aug-18	Amazon continues to burn in 2020 despite promises to save it	AP	https://apnews.com/article/ap-top-news-international-news-latin-america-science-56a278cf4b849dc06dadc309d06aa63f
2020-Aug-19	Bolsonaro says reports of Amazon fires are a ‘lie.’ Evidence says otherwise	CNN	https://edition.cnn.com/2020/08/19/americas/brazil-amazon-fires-bolsonaro-intl/index.html
2020-Aug-21	Merkel: Amazon deforestation threatens EU-Mercosur deal	DW	https://www.dw.com/en/merkel-amazon-deforestation-threatens-eu-mercosur-deal/a-54651194
2020-Aug-25	Climate crisis: New UK laws will clampdown on illegal deforestation in supply chains	Independent	https://www.independent.co.uk/climate-change/news/uk-law-illegal-deforestation-supply-chains-climate-crisis-amazon-a9686941.html
2020-Aug-28	Brazil, reversing course, says will keep fighting Amazon deforestation	Reuters	https://www.reuters.com/article/us-brazil-deforestation-idUSKBN25O2XP
2020-Sept-02	Amazon tribes are using drones to track deforestation in the Brazilian rainforest	CNN	https://www.cnn.com/2020/09/01/americas/amazon-drones-brazil-deforestation-cte-spc-intl/index.html
2020-Sept-02	Amazon tragedy repeats itself as Brazil rainforest goes up in smoke	The Guardian	https://www.theguardian.com/world/2020/sep/02/amazon-fires-brazil-rainforest-bolsonaro-destruction
2020-Sept-03	Brazil: Alarming number of new forest fires detected ahead of Amazon Day	Amnesty International	https://www.amnesty.org/en/latest/news/2020/09/brazil-alarming-number-of-new-forest-fires-detected-ahead-of-amazon-day/
2020-Sept-04	Brazil Fires Burn World's Largest Tropical Wetlands at ‘Unprecedented’ Scale	The New York Times	https://www.nytimes.com/2020/09/04/world/americas/brazil-wetlands-fires-pantanal.html
2020-Sept-09	Amazon: In the crosshairs of coronavirus and forest fires	BBC	https://www.bbc.com/news/world-latin-america-54071411
2020-Sept-10	In the Amazon, forest degradation is outpacing full deforestation	The Conversation	https://theconversation.com/in-the-amazon-forest-degradation-is-outpacing-full-deforestation-145901
2020-Sept-16	European nations warn Brazil over Amazon deforestation	DW	https://www.dw.com/en/european-nations-warn-brazil-over-amazon-deforestation/a-54954854
2020-Sept-17	Eight EU countries urge “real action” from Brazil to combat deforestation	MercoPress	https://en.mercopress.com/2020/09/17/eight-eu-countries-urge-real-action-from-brazil-to-combat-deforestation
2020-Sept-22	Bolsonaro to world: Brazil is victim of environmental smear	Washington Post	https://www.washingtonpost.com/world/the_americas/bolsonaro-to-world-brazil-is-victim-of-environmental-smear/2020/09/22/54505f2e-fce4-11ea-b0e4-350e4e60cc91_story.html
2020-Sept-30	Jair Bolsonaro attacks ‘international greed’ over Amazon – as it happened	The Guardian	https://www.theguardian.com/environment/live/2020/sep/30/xi-jinping-to-address-world-leaders-at-un-biodiversity-summit-live-updates-aoe
2020-Sept-30	Biden's criticism of Amazon deforestation draws swift reaction in Brazil	Reuters	https://www.reuters.com/article/usa-brazil-environment-idUSL1N2GR0WY
2020-Sept-30	Brazil's Bolsonaro slams Biden for ‘coward threats' over Amazon	Reuters	https://www.reuters.com/article/uk-usa-brazil-environment-idUKKBN26L2UE
2020-Oct-01	Fires in Brazil's Amazon worst in 10 years, data shows	Al Jazeera	https://www.aljazeera.com/news/2020/10/1/brazil-on-fire-amazon-blazes-worst-in-decade-data-shows
2020-Oct-01	Brazil's Amazon rainforest suffers worst fires in a decade	The Guardian	https://www.theguardian.com/environment/2020/oct/01/brazil-amazon-rainforest-worst-fires-in-decade
2020-Nov-13	Deforestation in Brazil's Amazon rises for first time in four months	Reuters	https://www.reuters.com/article/us-brazil-environment-idUSKBN27T1X2
